# Social stigma is an underestimated contributing factor to unemployment in people with mental illness or mental health issues: position paper and future directions

**DOI:** 10.1186/s40359-020-00399-0

**Published:** 2020-04-21

**Authors:** Evelien P. M. Brouwers

**Affiliations:** Scientific Center for Care and Wellbeing, Tilburg School of Social and Behavioral Sciences, Tilburg University, P.O. Box 90153, 5000 LE Tilburg, The Netherlands

**Keywords:** Social stigma, Mental health, Employment, Discrimination, Disclosure, Common mental disorders, Severe mental disorders, Workplace, Attitudes, Treatment gap

## Abstract

**Background:**

As yet, little is known about the effects of mental health stigma on sustainable employment. This is surprising, as mental health stigma is common, and because people with severe and common mental disorders are 7 and 3 times more likely to be unemployed, respectively, than people with no disorders. As the global lifetime prevalence of mental disorders is 29%, the high unemployment rates of people with these health problems constitute an important and urgent public health inequality problem that needs to be addressed.

**Main text:**

The aim of this position paper is to illustrate the assumption that stigma contributes to the unemployment of people with mental illness and mental health issues with evidence from recent scientific studies on four problem areas, and to provide directions for future research. These four problem areas indicate that: (1) employers and line managers hold negative attitudes towards people with mental illness or mental health issues, which decreases the chances of people with these health problems being hired or supported; (2) both the disclosure and non-disclosure of mental illness or mental health issues can lead to job loss; (3) anticipated discrimination, self-stigma and the ‘Why Try’ effect can lead to insufficient motivation and effort to keep or find employment and can result in unemployment; and (4) stigma is a barrier to seeking healthcare, which can lead to untreated and worsened health conditions and subsequently to adverse occupational outcomes (e.g. sick leave, job loss).

**Conclusions:**

The paper concludes that stigma in the work context is a considerable and complex problem, and that there is an important knowledge gap especially regarding the long-term effects of stigma on unemployment. To prevent and decrease adverse occupational outcomes in people with mental illness or mental health issues there is an urgent need for high quality and longitudinal research on stigma related consequences for employment. In addition, more validated measures specifically for the employment setting, as well as destigmatizing intervention studies are needed.

## Background

### Unemployment and mental health are strongly associated

Previous research has shown that people with severe and common mental disorders are 7 and 3 times more likely to be unemployed, respectively, than people with no disorders [1]. Generally, job loss has been shown to lead to decreases in health, and reemployment after unemployment has been shown to lead to increased health [2, 3]. In particular, people with mental disorders could benefit from the positive aspects of employment [4] but have difficulties finding and keeping employment. This poses a public health inequality problem, as mental disorders are highly prevalent: the lifetime prevalence of mental disorders in the global population is 29% [5]. Mental disorders are even more prevalent in young people: in OECD countries, approximately one in four 15 to 24 year-olds have a mental disorder, which puts them at a higher risk of dropping out of school and having poorer chances of finding stable employment [1]. A recent epidemiological study investigating the influence of different chronic health problems on loss of employment in workers aged 45–64 showed that workers with self-reported psychological health problems had the highest risk of unemployment. Moreover, compared to workers with other chronic health conditions, they also had a higher risk of other adverse occupational outcomes, such as taking early retirement and exiting the work force via disability benefits and sick leave [6–8]. These findings are noteworthy and alarming, as they illustrate that not only mental *illness*, but also much milder mental health issues can have adverse effects on employment outcomes. Sick leave has been found to be a predictor of future sick leave, unemployment and disability pension [9]. Unemployment is associated with poverty, and the societal costs of lost productivity due to ill mental health are enormous [10, 11]. In sum, it is important to find solutions to this societal problem and improve the *sustainable* employment of both groups, i.e. people with mental health illness as well as people with mental health issues (hereafter referred to as ‘MI/MHI’).

## Main text

### The knowledge gap of stigma in the work context

**Fig. 1:**
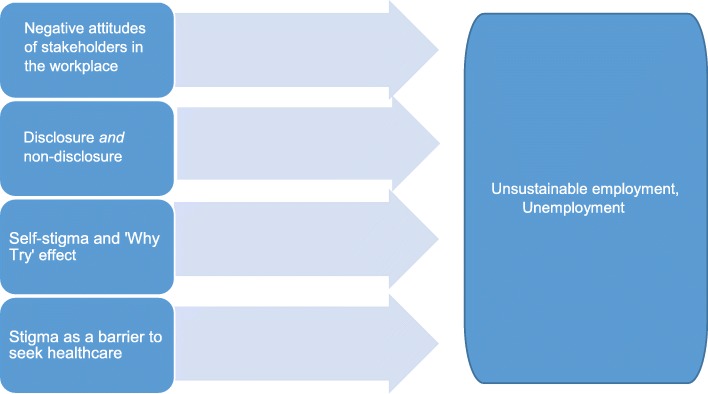
Four key problem areas of social stigma for sustainable employment of people with mental illness or mental health issues

In the literature on absenteeism and return to work in people with common mental disorders, the role of workplace stigma is largely ignored. In contrast, in the *stigma* literature, the number of studies on the work environment is growing, but the main focus is rarely on long-term employment outcomes. Hence, there is an important knowledge gap regarding the prevention of job loss and other adverse work outcomes related to stigma in a large group that (globally) holds a vulnerable position in the labor market. There is an urgent need for high-quality and longitudinal research that investigates the role of stigma in the prevention of job loss in people with MI/MHI. In this paper, I argue that social stigma is an important underestimated contributing factor to unemployment in people with MI/MHI. I illustrate my views with evidence from recent scientific studies on four problem areas that support the assumption that stigma leads to unemployment, and I provide directions for future research. These four areas are depicted in Fig. 1.

### Social stigma defined

Before discussing the impact of stigma on employment, it is essential to address what stigma actually is. The word *stigma* means *burn* in Greek and refers to the time when slaves and criminals were marked with a burn to show others that this person was to be avoided [12]. An influential definition of stigma comes from Link and Phelan [13] who describe stigma as the co-occurrence of several components (1) *distinguishing and labeling* differences between people (e.g. “People with or without MI/MHI are different”); (2) *linking these differences to negative stereotypes* (e.g. “People with MI/MHI often are dangerous”); (3) *separating “us” from “them*” (e.g. “Workers with MI/MHI are less competent than us without MI/MHI”) and (4) *Status loss and discrimination* (e.g. “A job applicant with MI/MHI is perceived as less competent and therefore not hired”). The authors also stress that for stigmatization to occur, there must be a difference in power between the stigmatizer and the stigmatized (e.g. an employer versus a job applicant with MI/MHI). Stigma exists at different levels. *Individual stigma* refers to the psychological processes in which individuals engage in responses to stigma, such as self-stigma. *Interpersonal stigma* refers to interactions that occur between the stigmatized and the non-stigmatized, and *structural stigma* refers to a structural restriction of the opportunities of stigmatized groups, for instance by law, or institutional policies [14]. Finally, it has been suggested that stigmatization is a process, resulting from inadequate or insufficient knowledge [15]. Although mental health stigma has been extensively researched among the general population, less is known about its prevalence and consequences in the workplace [16]. Consequently, there also is an important knowledge gap regarding the prevention of job loss and other adverse work outcomes related to stigma. There are -at least- four areas that warrant further study and that will now be addressed.

### Four problem areas through which stigma can lead to unemployment and other adverse occupational outcomes (e.g. sick leave, disability benefits and early retirement)

*First problem area: Employers and other stakeholders in the work environment often hold negative attitudes towards people with MI/MHI which decreases the chances of people with MI/MHI being hired or supported.*

A first problem is that employers often hold negative attitudes towards people with MI/MHI, which may negatively affect hiring decisions and supportive supervisor behavior. Reported employer beliefs include that workers with MI/MHI lack the competence to meet the demands of work, that they need supervision, and that working is not healthy for them [17, 18]. While in *some individual* cases such assumptions may be true, it is problematic if employers see people with MI/MHI as a *group* that is characterized by these negative stereotypes, rather than as individuals. Employers have been found to hold negative attitudes not only towards people who *currently have* MI/MHI, but also towards people who have had MI/MHI in the past [19]. Hence, even when health problems have disappeared, the negative label (stigma) continues to exist. Across different countries and cultures, people with mental illness indicate that workplace discrimination is a prevalent problem [20].

A particularly harmful negative stereotype against people with mental illness is that they are dangerous or unpredictable [18, 21]. This stereotype may strongly be influenced by media coverage and is heavily endorsed by television series, movies and novels [22, 23] in which this stereotype is useful for an interesting plot. Studies have consistently shown that both entertainment and news media provide overwhelmingly dramatic and distorted images of mental illness that emphasize dangerousness, criminality and unpredictability, and employers and supervisors are exposed to these influences as well. A recent review concluded that destigmatizing interventions targeting media professionals should be a research priority [24]. Entertainment and news media model negative reactions to people with mental illness, including fear, rejection, derision and ridicule [23]. Indeed, employers have reported negative beliefs such as that it would be unsafe to let people with mental illness work with vulnerable people such as children and the elderly and that they are likely to be injured; are unable to deal with money; and cannot be trusted handling confidential information including credit cards, names and addresses [17]. A survey of 500 British employers showed that 32% believed that organizations take a significant risk when employing people with mental health problems in a public or client facing role [25]. A high number of employers (72%) have been found to believe that potential employees should disclose MI/MHI prior to recruitment [25]. However, studies have shown that if employers are aware of job applicants’ mental health conditions, they are less inclined to hire them [26, 27].

There are also other important workplace stakeholders whose stigmatizing attitudes can negatively affect the employment opportunities of people with MI/MHI. For instance, a recent Dutch multi-stakeholder focus group study on the disclosure of MI/MHI in the work environment suggested that human resource staff in particular were in favor of job applicants’ disclosure because it allowed them to identify and discriminate people with MI/MHI and avoid financial risk, which they viewed it as a core responsibility of their jobs [28]. Although this was a qualitative study and no conclusions can be drawn regarding generalizability of the findings, the authors emphasize that this finding warrants further quantitative study. Another stakeholder group are mental health providers. They can play an important role in helping clients with mental illness gain and maintain employment by referring them to supported employment programs. However, a main barrier to referring clients to supported employment programs is their expectation that clients will be discriminated against at work, and that they do not view employment or self-sufficiency as important factors in their clients’ recovery [29]. Hence, whereas there are evidence-based and effective supported employment programs, healthcare providers’ behavior can harm their clients’ chances of employment. In conclusion, these findings suggest that structural stigma and discrimination of people with MI/MHI by workplace stakeholders is a considerable problem that increases the risk of long-term unemployment.

More research is needed on the knowledge, attitudes and behavior of workplace stakeholders and on what they need to adequately support rather than exclude people with MI/MHI in the work environment. For instance, supervisors may lack knowledge of how to handle mental health issues and appropriately support workers with MI/MHI [30]. Although there is some evidence that interventions improving *public* knowledge about mental illness can be effective in reducing stigma [31], much less is known about *how to improve employers’* knowledge and attitudes. Additionally, whereas some studies have shown that training managers in workplace mental health can improve their knowledge, attitudes and self-reported behavior in supporting employees experiencing MI/MHI [19, 32], it is less clear what the exact content of such training should consist of [19].

Due to a lack of good-quality studies, it is unknown what impact manager training has on employee well-being [19] and long term employment. Future destigmatizing intervention studies targeting employers are needed to support line managers in showing inclusive leadership and thereby decreasing vulnerability of people with MI/MHI in the labor market.

*Second problem area: Both the disclosure and nondisclosure of MI/MHI in the work environment can lead to job loss.*

Due to the fear of being stigmatized, many workers conceal their MI/MHI in the work environment. For example, in a Canadian study, a random sample of 2219 workers were asked if they would disclose a mental health problem in the workplace. One-third of the workers said they would not tell their managers if they experienced a mental health problem, mostly due to a fear of damaging their careers [33]. Similarly, another study showed that military personnel feared that disclosure of a mental health difficulty would not be held in confidence and would therefore act as a structural barrier for career progression [34]. The findings of these studies illustrate that workplace discrimination is perceived not only by people with MI/MHI themselves [21] but also by others.

However, disclosure can also be crucial to *prevent* adverse occupational outcomes such as sick leave and job loss. If important stakeholders in the work environment (e.g. line managers) know what the employee’s needs are, they can provide practical and emotional support, which can in turn help the employee to stay at work. A Dutch study found that as many as 43% of workers with MI/MHI indicated a need for work adjustments to stay at work, and that work adjustments were associated with decreased sick leave [35]. However, to provide workplace support, workplace stakeholders need to be informed about employees’ needs. Nondisclosure can therefore result in employees missing out on support that may be available and that can help them to stay at work.

Another advantage of disclosure is that it enables authenticity (i.e., employees’ ability to act in accordance with their true selves) [27, 36] in the work environment. Authenticity has been shown to be positively related to work engagement, job satisfaction and work performance [36]. Important reasons for the disclosure of MI/MHI are that people feel a need to be honest, want to explain their own behavior and find concealment stressful [37].

In summary, disclosure can *prevent* adverse outcomes such as job loss as it can lead to work adjustments, social support and authenticity. Simultaneously, it can also *lead* to job loss through stigma and discrimination. This suggests that disclosure decisions are highly complex and need to be made deliberately. Two small-scale studies indeed found that supporting unemployed people with mental illness in managing their personal health information led to significantly higher employment rates after 6–12 weeks [38, 39]. However, larger-scale and longitudinal intervention studies investigating the effect of decisional support in people with MI/MHI are lacking and are urgently needed. Additionally, more fundamental research is needed for theory building to better understand the decision-making process.

*Third problem area: anticipated discrimination, self-stigma and the ‘Why Try’ effect can lead to insufficient motivation and effort to keep or find employment.*

Several studies have illustrated that anticipated discrimination and self-stigma discourage people with MI/MHI from undertaking relevant employment-related actions. For instance, an international study of people with depression found that 25% had stopped applying for work and that 20% had stopped applying for education or training *because of anticipated stigma and discrimination* [40]. Similarly, two other studies indicated that 64% of people with schizophrenia and 39% of people with substance use disorders had stopped applying for work, training or education because of anticipated discrimination [41, 42]. Many people with MI/MHI experience discrimination or expect to be discriminated in the work environment because of their health problems. For instance, in a study on major depression including respondents from 35 countries, over 60% had experienced or anticipated discrimination in the work setting. Moreover, in the very high developed countries, almost 60% of respondents had stopped themselves from applying for work, education or training because of anticipated discrimination [20].

Self-stigma occurs when people with MI/MHI are aware of, agree with and apply existing negative stereotypes to themselves [43]. The application of negative stereotypes to oneself has been described to lead to two types of consequences. First, self-stigma can lead to emotional consequences, such as diminished self-respect, disempowerment, feelings that one is unworthy or incapable in achieving personal goals. Second, it may lead to behavioral consequences, also known as the “Why try” effect. Here, as a consequence of self-stigma, people refrain from the performance of important behavior, such as proactively trying to keep their employment or by actively trying to find new employment. Hence, the “Why try” effect is a behavioral intention, or perhaps more accurately, a behavioral dis-intention [43]. If people with MI/MHI make insufficient effort to find or keep employment as a result of self-stigma, this increases their risk of unemployment. Whereas cognition is increasingly gaining attention in work rehabilitation research, such as research on return-to-work perceptions and return-to-work self-efficacy [44–46], studies on the long-term effects of self-stigma, anticipated discrimination and especially on the “Why Try” effect on unemployment are scarce and warrant more attention [47]. Moreover, considering the high prevalence of the “Why Try” effect, more intervention studies on how to overcome self-stigma and improve work-related self-efficacy are urgently needed.

*Fourth problem area: Stigma is a barrier to seeking healthcare, which can lead to untreated and worsened health conditions and subsequently to unemployment*.

A fourth reason why stigma acts as a barrier to sustained employment is that it can prevent people with MI/MHI from seeking available healthcare, due to a fear of being treated differently in the work environment. Research with professionals with a high risk for trauma exposure, such as journalists, police officers, railway workers and soldiers has frequently shown that seeking help for MI/MHI is inhibited by concerns about the potentially negative views of colleagues and managers [48–52]. For instance, a study of police officers showed that whereas PTSD, depression and alcohol abuse were common, most had never sought mental health services. Their most commonly cited barriers to accessing services were concerns regarding confidentiality and the potential “negative career impact” [49]. Similarly, a recent systematic review showed that approximately 60% of military personnel who experience MI/MHI do not seek help, even though many could benefit from professional treatment [51]. The low use of mental healthcare by military personnel may be due to a number of factors, although across military studies, one of the most frequently reported barriers to help-seeking for MI/MHI is concerns about stigma [52, 53]. For instance, a study among US Marines indicated that they thought that receiving psychiatric services would cause them to be seen as weak and that 64% believed they would be treated differently by their unit leaders if they sought help [51]. Another recent systematic review showed a substantial negative relationship between stigma and help seeking for mental health difficulties within the armed forces [34]. Missing out on professional treatment poses a risk for worsening health problems, sick leave, loss of employment, and substantial associated costs. In conclusion, while many studies have shown that workers often avoid seeking health care due to a fear of stigma and discrimination, studies on how to overcome this problem and especially the consequences of this problem for long-term employment outcomes are scarce. Again, more fundamental as well as intervention research is needed to better understand the decision-making process and to evaluate the effects of intervention studies.

## Conclusions

### Final remarks

Four problem areas were discussed that illustrate why stigma is a contributing factor to the more vulnerable labor market position of people with MI/MHI. In this paper, both people with mild mental health issues and people with severe mental illness were taken together as one group, which does not do justice to the wide variety in individual differences that exists among this large part of the population. Moreover, taking these groups together ignores the fact that the degree of stigmatization may vary depending on the type of diagnosis, and on symptom severity [28]. However, these groups were taken together to make an essential point in this paper, i.e. that in both people with *severe mental illness* as in people with *mild mental health issues* stigma is likely to lead to adverse job outcomes, and that more research in this area is urgently needed.

Whereas this position paper specifically addressed *mental health* stigma, the four problem areas that were examined are also likely to hamper the sustainable employment of other stigmatized groups. For example, recently it has been reported that people with concealable physical illnesses such as diabetes [54], HIV [55] or a history of cancer [56] often face the same dilemma of whether or not to disclose their health problems in the workplace. These findings underline that it often is stakeholders’ attitudes rather than the worker’s illness that is the problem for sustainable employment. Across different contexts, illnesses and situations, more research on stigma and its long-term effects on employment are needed.

In conclusion, research is increasingly showing that stigma in the work context is a considerable and complex problem and that theoretical knowledge in this area is scarce but much needed for the development of effective interventions. To date, few rigorous destigmatizing intervention studies have been conducted. Especially studies with a focus on long-term employment outcomes are lacking, which is surprising considering the scope of this public health problem. Also, more studies on structural stigma, and on interventions to gain insight in how to decrease structural stigma (e.g. policy reviews) are needed. Moreover, there is currently a limited availability of validated measures of mental health stigma that have been contextualized to the workplace setting [57], which are a prerequisite for high-quality research. It is important that specific measures for the work context be developed, as specific measures will better enable the evaluation of the consequences of stigma for long-term (un) employment than more general stigma measures. Considering the high prevalence of MI/MHI, the achievement of stigma-free work environments with a focus on people’s needs to perform and be well, rather than on concealment, stigma, and discrimination, would be beneficial for individuals, employers and society at large.

## Data Availability

Not applicable.

## Abbreviation

MI/MHI: Mental Illness or Mental Health Issues

## References

[1] OECD (2012). Sick on the job? Myths and Realities about Mental Health and Work.

[2] Schuring M, Mackenbach J, Voorham T, Burdorf A (2011). The effect of re-employment on perceived health. J Epid Comm Health.

[3] Schuring M, Burdorf A. The benefits of paid employment among persons with common mental health problems: evidence for the selection and causation mechanism. Scand J Work Environ Health. 2017. 10.5271/sjweh.3675. 10.5271/sjweh.3675 28967666

[4] Van der Noordt M, IJzelenberg H, Droomers M, Proper K (2014). Health effects of employment: a systematic review of prospective studies. Occup Environ Med.

[5] Steel Z, Marnane C, Iranpour C, Chey T, Jackson JW, Patel V (2014). The global prevalence of common mental disorders: a systematic review and meta-analysis. Int J Epidemiol.

[6] Leijten F, De Wind A, van den Heuvel SG, Ybema JF, Van der Beek AJ, Robroek SJ (2015). The influence of chronic health problems and work-related factors on loss of paid employment among older workers. J Epid Comm Health..

[7] Leijten F, van den Heuvel SG, Ybema JF, Robroek SJW, Burdorf A (2013). Do work factors modify the association between chronic health problems and sickness absence among older employees? Scand J work. Environ Health.

[8] Luciano A, Meara E (2015). Employment status of people with mental illness: national survey data from 2009 and 2010. Psych Serv.

[9] Hultin H, Lindholm C, Malfert M, Möller J. Short-term sick leave and future risk of sickness absence and unemployment- the impact of health status. BMC Public Health. 2012. 10.1186/1471-2458-12-861. 10.1186/1471-2458-12-861 PMC3508966 23050983

[10] Hewlett E, Moran V (2014). Making Mental Health Count: The Social and Economic Costs of Neglecting Mental Health Care.

[11] Chisholm D, Sweeny K, Sheehan P, Rasmussen B, Smit F, Cuijpers P, et al. Scaling-up treatment of depression and anxiety: a global return on investment analysis. Lancet Psychiatry. 2016. 10.1016/%20S2215-0366(16)30024-4. 10.1016/S2215-0366(16)30024-4 27083119

[12] Finzen A. Stigma and Stigmatization Within and Beyond Psychiatry. In: Gaebel W., Rössler W., Sartorius N. (eds) The Stigma of Mental Illness - End of the Story?. Cham: Springer; 2017.

[13] Link BG, Phelan JC. Conceptualizing Stigma. Ann Rev Sociol. 2001. 10.1146/annurev.soc.27.1.363.

[14] Hatzenbuehler ML (2016). Structural stigma: Research evidence and implications for psychological science. Am Psychol.

[15] Thornicroft G, Rose D, Kassam A, Sartorius N (2007). Stigma: ignorance, prejudice or discrimination?. Br J Psychiatry.

[16] Hanisch SE, Twomey CD, Szeto AC, Birner UW, Nowak D, Sabariego C. The effectiveness of interventions targeting the stigma of mental illness at the workplace: a systematic review. BMC Psychiatry. 2016;16:1. 10.1186/s12888-015-0706-4. Published 2016 Jan 6. 10.1186/s12888-015-0706-4 PMC4704270 26739960

[17] Biggs D, Hovey N, Tyson P, MacDonald S. Employer and employment agency attitudes towards employing individuals with mental health needs. J Ment Health. 2010. 10.3109/09638237.2010.507683. 10.3109/09638237.2010.507683 20874510

[18] Krupa T, Kirsch B, Cockburn L, Gewurtz R (2009). Understanding the stigma of mental illness in employment. Work..

[19] Gayed A, Milligan-Saville J, Nicholas J, Bryan BT, LaMontagne AD, Milner A, et al. Effectiveness of training workplace managers to understand and support the mental health needs of employees: a systematic review and meta-analysis. Occup Environ Med. 2018. 10.1136/oemed-2017-104789. 10.1136/oemed-2017-104789 29563195

[20] Brouwers EPM, Matthijssen J, Van Bortel T, Knifton L, Wahlbeck K, Audehove V, et al. Discrimination in the workplace, reported by people with major depressive disorder: a cross-sectional study in 35 countries. BMJ Open. 2016. 10.1136/bmjopen-2015-009961. 10.1136/bmjopen-2015-009961 PMC4769412 26908523

[21] Economou M, Peppou LE, Souliotis K, Lazaratou H, Kontoangelos SN, et al. Attitudes to depression and psychiatric medication amid the enduring financial crisis in Attica: Comparison between 2009 and 2014. Int J Social Psychol. 2019. 10.1177/0020764019858653. 10.1177/0020764019858653 31250687

[22] Arboleda-Flórez J, Stuart H (2012). From sin to science: fighting the stigmatization of mental illnesses. Can J Psychiatr.

[23] Stuart H (2006). Media portrayal of mental illness and its treatments: what effect does it have on people with mental illness?. CSN Drugs.

[24] Ross AM, Morgan AJ, Jorm AD, Reavley NJ (2019). A systematic review of the impact of media reports of severe mental illness on stigma and discrimination, and interventions that aim to mitigate any adverse impact. Soc Psychiatr Psychiatr Epidem..

[25] Henderson C, Williams P, Little K, Thornicroft G (2013). Mental health problems in the workplace: changes in employers’ knowledge, attitudes and practices in England 2006-2010. Br J Psych Suppl.

[26] Hipes C, Lucas J, Phelan J, White R. The stigma of mental illness in the labor market. Soc Sci Res. 2016. 10.1016/j.ssresearch.2015.12.001. 10.1016/j.ssresearch.2015.12.001 26857169

[27] Rüsch N, Corrigan P, Waldmann T, Staiger T, Bahemann A, Oexle N (2018). Attitudes toward disclosing a mental health problem and reemployment: a longitudinal study. J Nerv Ment Dis.

[28] Brouwers EPM, Joosen MCW, van Zelst C, et al. To disclose or not to disclose: A multi-stakeholder focus group study on mental health issues in the work environment. J Occup Rehabil. 2019. 10.1007/s10926-019-09848-z. 10.1007/s10926-019-09848-z PMC7031172 31410722

[29] Costa M, Baker M, Davidson L, Giard J, Guillorn L, Gonzalez Ibanez A (2017). Provider perspectives on employment for people with serious mental illness. Int J of Social Psychiatry.

[30] Kirsch B, Krupa T, Luong D (2018). How do supervisors perceive and manage employee mental health issues in their workplaces?. Work..

[31] Gronholm PC, Henderson C, Deb T, Thornicroft G (2017). Interventions to reduce discrimination and stigma: the state of the art. Soc Psychiatr Psychiatr Epidem.

[32] Ellis AM, Casey TW, Krauss AD (2017). Setting the foundation for well-being: evaluation of a supervisor-focused mental health training. Occup Health Sci.

[33] Dewa C (2014). Worker attitudes towards mental health problems and disclosure. Int J Occup Environ Med.

[34] Coleman S, Stevelink S, Hatch S, Denny J, Greenberg N (2017). Stigma-related barriers and facilitators to help seeking for mental health issues in the armed forces: a systematic review and thematic synthesis of qualitative literature. Psych Med.

[35] Boot C, Van den Heuvel S, Bültmann U, De Boer A, Koppes L, Van der Beek AJ (2013). Work adjustments in a representative sample of employees with a chronic disease in the Netherlands. J Occupat Rehabil.

[36] Metin B, Taris T, Peeters M, Van Beek I, Van den Bosch R (2016). Authenticity at work – a job-demands resources perspective. J Man Psych.

[37] Brohan E, Henderson C, Wheat K, Malcolm E, Clement S, Barley E (2012). Systematic review of beliefs, behaviours and influencing factors associated with disclosure of a mental health problem in the workplace. BMC Psychiatry.

[38] Henderson C, Brohan E, Clement S, Williams P, Lassman F, Schauman O (2013). Decision aid on disclosure of mental health status to an employer: feasibility and outcomes of a randomised controlled trial. Br J Psych.

[39] McGahey E, Waghorn G, Lloyd C, Morrissey S, Williams P (2016). Formal plan for self-disclosure enhances supported employment outcomes among young people with severe mental illness. Early Interv Psychiatr.

[40] Lasalvia A, Zoppei S, Van Bortel T, Bonetto C, Christofalo D, Wahlbeck K (2013). Global pattern of experienced and anticipated discrimination reported by people with depressive disorder: a cross-sectional survey. Lancet..

[41] Thornicroft G, Brohan E, Rose D, Sartorius N, Leese M (2009). Global pattern of anticipated and experienced discrimination against people with schizophrenia: a cross-sectional survey. Lancet.

[42] van Boekel L, Brouwers E, Van Weeghel J, Garretsen H. Experienced and anticipated discrimination reported by individuals in treatment for substance use disorders within the Netherlands. Health Soc Care Comm. 2016. 10.1111/hsc.12279. 10.1111/hsc.12279 26417904

[43] Corrigan P, Bink A, Schmidt A, Jones N, Rusch N (2016). What is the impact of self-stigma? Loss of self-respect and the “why try” effect. J Ment Health.

[44] Nieuwenhuijsen K, Noordik E, Van Dijk FJ, Van der Klink JJL. Return to work perceptions and actual return to work in workers with common mental disorders. J Occup Rehab. 2012:290–9. 10.1007/s10926-012-9389-6 23124685

[45] Van Beurden K, Van der Klink JJL, Brouwers EPM, Joosen MCW, Mathijssen J, Terluin B (2015). Effect of an intervention to enhance guideline adherence of occupational physicians on return-to-work self-efficacy in workers sick-listed with common mental disorders. BMC Public Health.

[46] Volker D, Zijlstra-Vlasveld M, Brouwers EPM, Van Lomwel A, Van der Feltz-Cornelis C (2015). Return-to-work self-efficacy and actual return to work among long-term sick-listed employees. J Occup Rehab..

[47] Stuart H, Chen SP, Christie R, Dobson K, Kirsh B, Knaak S (2014). Opening minds in Canada: targeting change. Can J Psychiatr.

[48] Fox J, Desai M, Britten K, Lucas G, Luneau R, Rosenthal M (2012). Mental-health conditions, barriers to care, and productivity loss among officers in an urban police department. Conn Med.

[49] Greenberg N, Gould M, Langston V, Brayne M (2009). Journalists’ and media professionals’ attitudes to PTSD and help-seeking: a descriptive study. J Ment Health.

[50] Sage C, Brooks S, Jones N, Greenberg N (2016). Attitudes towards mental health and help-seeking in railway workers. Occup Med.

[51] Sharp ML, Fear NT, Rona RJ, Wesseley S, Greenberg N, Jones N (2015). Stigma as a barrier to seeking health care among military personnel with mental health problems. Epidem Rev.

[52] Williamson V, Greenberg N, Stevelink SAM. Perceived stigma and barriers to care in UK Armed Forces personnel and veterans with and without probable mental disorders. BMC Psychol. 2019. 10.1186/s40359-019-0351-7. 10.1186/s40359-019-0351-7 PMC6881983 31775853

[53] Vermetten E, Greenberg N, Boeschoten M, Delahaije R, Jetley R, Castro C. Deployment-related mental health support: comparative analysis of NATO and allied ISAF partners. Eur J Traumat. 2014. 10.3402/ejpt.v5.23732. 10.3402/ejpt.v5.23732 PMC4138710 25206953

[54] Hakkarainen P, Munir F, Moilanen L, Räsänen K, Hänninen V. Concealment of type 1 diabetes at work in Finland: a mixed-method study. BMJ Open. 2018. 10.1136/bmjopen-2017-019764. 10.1136/bmjopen-2017-019764 PMC5781107 29331976

[55] Stutterheim S, Brands R, Baas I, Lechner L, Kok G, Bos A (2017). HIV status disclosure in the workplace: positive and stigmatizing experiences of health care workers living with HIV. J Assoc Nurs AIDS Care.

[56] Stergiou-Kita M, Pritlove C, Kirsch B (2016). The “big C”-stigma, cancer, and workplace discrimination. J Cancer Surv.

[57] Martin AJ, Giallo R (2016). Confirmatory factor analysis of a questionnaire measure of managerial stigma towards employee depression. Stress Health.

